# Impact of Activation of *EGFL7* within Microenvironment of High Grade Ovarian Serous Carcinoma on Infiltration of CD4+ and CD8+ Lymphocytes

**DOI:** 10.3390/medicina58050588

**Published:** 2022-04-24

**Authors:** Jacek J. Sznurkowski, Anton Żawrocki, Natalia Krawczyńska, Michał Bieńkowski, Bartosz Wasąg, Wojciech Biernat

**Affiliations:** 1 Department of Oncological Surgery, Medical University of Gdansk, Smoluchowskiego 17, 80-214 Gdansk, Poland; 2 Department of Pathomorphology, Medical University of Gdansk, 80-214 Gdansk, Poland; zawrocki@gumed.edu.pl (A.Ż.); michal.bienkowski@gmail.com (M.B.); biernat@gumed.edu.pl (W.B.); 3 Department of Biology and Medical Genetics, Medical University of Gdansk, 80-210 Gdansk, Poland; natalia.krawczynska@gumed.edu.pl (N.K.); bartosz.wasag@gumed.edu.pl (B.W.); 4 Laboratory of Clinical Genetics, University Clinical Centre, 80-952 Gdansk, Poland

**Keywords:** ovarian cancer, Egfl-7, ICAM-1, CD4+, CD8+, diapedesis

## Abstract

*Background*: It has been demonstrated that Egfl7 promotes tumor cell escape from immunity by downregulating the activation of tumor blood vessels. *Aim*: to analyze mRNA expression of *EGFL7* within the tumor microenvironment of high-grade ovarian serous carcinoma and its association with a number of intraepithelial CD4+/CD8+ lymphocytes and ICAM-1 expression. *Methods:* qPCR analysis of *EGFL7* mRNA in cancer cells and adjacent stromal endothelium microdissected from formalin-fixed paraffin-embedded tumors of 59 high-grade ovarian serous carcinoma patients, was performed. Infiltration of intraepithelial lymphocytes (CD4+/CD8+) and expression of ICAM-1 were evaluated by immunohistochemistry and compared between tumors with different statuses of *EGFL7* expression. *Results*: *EGFL7* was expressed in cancer cells (9/59, 15.25%), endothelium (8/59, 13.56%), or both cancer cells and adjacent endothelium (4/59, 6.78%). ICAM-1 was expressed on cancer cells (47/59, 79.66%), stromal endothelium (46/59, 77.97%), or both epithelium and endothelium (40 of 59, 67.8%). *EGFL7*-positivity of cancer cells and endothelium was associated with lower intraepithelial inflow of CD4+ (*p* = 0.022 and *p* = 0.029, respectively) and CD8+ lymphocytes (*p* = 0.004 and *p* = 0.031, respectively) but impact neither epithelial nor endothelial ICAM-1 expression (*p* = 0.098 and *p* = 0.119, respectively). The patients’ median follow-up was 23.83 months (range 1.07–78.07). Lack of prognostic significance of *EGFL7*-status and ICAM-1 expression was notified. *Conclusion*: *EGFL7* is activated in the cancer cells as frequently as in the endothelium of human high-grade ovarian serous carcinoma. Activation of *EGFL7* in cancer cells and/or endothelial cells could negatively impact diapedesis regardless of localization.

## 1. Background

Ovarian cancer (OvCa) is the leading cause of death from gynecological malignancy in the developed world. The overall five-year survival rate for patients with advanced OvCa is only 30–40% [1]. Notwithstanding the good initial response to primary therapy, around 80% of the patients with advanced disease will develop recurrence and finally succumb to the illness [1]. Therefore, more insight into the interaction between OvCa and the immune system is needed to develop more effective anti-tumor immunotherapy to improve the clinical outcome.

Instead of being targeted for immune destruction, OvCa has the ability to escape immune surveillance by creating a highly suppressive environment [2]. Tumor escape from immunity is undoubtedly a promising phenomenon to consider while designing therapeutic tools aimed at preventing cancer progression and metastasis [3,4].

Blood vessels, which are a part of the tumor microenvironment, constitute a natural physical barrier that regulates the immune response via diapedesis [5]. Activated endothelial cells express a high level of cell surface leukocyte adhesion molecules, such as E- and P-selectin, intercellular adhesion molecule-1 (ICAM-1), and vascular cell adhesion molecule-1 (VCAM-1) which participate in the capture of circulating immune cells through the rolling, arrest, firm adhesion, and extravasation of leukocytes into stroma [5,6]. Thus, immune escape may be achieved by limiting the influx of immune effectors into cancer tissue [7,8] through down-regulation of endothelial adhesion molecules ICAM-1 and VCAM-1 [9,10,11,12].

EGFL7 is an endothelial cell-derived protein that regulates vascular tube formation. Its expression is downregulated in mature vasculature, upregulated in proliferating endothelium [13,14,15] and deregulated in human cancer [16].

Using experimental approaches, it has been demonstrated that, when expressed by cancer cells, *EGFL7* promotes tumor escape from immunity by downregulating the activation of tumor blood vessels [17]. EGFL7 in mouse breast and lung carcinoma cells inhibits the endothelial expression of ICAM-1 and VCAM-1, preventing lymphocyte adhesion and transendothelial migration [17]. This observation was additionally confirmed by an analysis of tissue samples derived from a large cohort of breast cancer patients, utilizing hybridization in situ as a method to localize a real source of EGFL7 secretion [18].

Since *EGFL7* is a plasma membrane-associated sialidase (lysosomal sialidase) that could accumulate at distant sites from the producing cells [15], it is impossible to answer if the elevated expression of *EGFL7* transcripts observed in several human cancer tumors come from activated cancer epithelium or up-regulated endothelial cells of the surrounding stroma.

Laser Capture Microdissection (LCM) technology enables harvesting the cells of interest directly or can isolate specific cells by cutting away unwanted cells to give histologically pure enriched cell populations [19].

The aim of this study was to analyze the expression of *EGFL7* within the cancer epithelial nest and the adjacent stroma (tumor microenvironment) of High Grade Ovarian Serous Carcinomas (HGOSCs) and its impact on the number of immune cell effectors (CD8+, CD4+) infiltrating cancer epithelial nests, as well as correlation with ICAM-1 expression of adjacent blood vessels.

## 2. Material and Methods

### 2.1. Human Samples

A cohort of 59 HGOSC patients was treated with primary debulking surgery (PDS) at the Department of Gynecologic Oncology, Oncological Center of Gdynia, Polish Red Cross Hospital Gdynia (Poland) between September 2013 and December 2015. 

The patients were informed that surgical specimens would be used for conventional pathological diagnosis and that the remaining tissues would be used for research. No personal patient data was required for this study and the protocols carried no risk, so only verbal informed consent was required from the patients. The protocol was approved by the Ethics Committee of Medical University of Gdansk (Permit Number: NKBBN/263/2012). Disease staging was defined according to the International Federation of Gynecology and Obstetrics (FIGO) staging system [20]. 

### 2.2. Tissue Samples

We analyzed 59 formalin-fixed paraffin-embedded (FFPE) tissue samples from primary tumors for immunohistochemical (IHC) evaluation of expression of ICAM-1, CD4 and CD8 and for qPCR analysis of Egfl7 mRNA transcripts.

### 2.3. Immunohistochemistry

The immunohistochemical stainings were performed on whole sections of representative FFPE blocks. The staining was performed according to the following protocol (antibody details, suppliers and dilutions are listed in Table 1). 

Four µm-thick serial sections were cut, deparaffinized and subjected to a heat-induced epitope retrieval step before being incubated with the primary antibodies. Sections were immersed in Target Retrieval Solution (pH 6.0; Dako Cytomation, Denmark) and heated in a pressure cooker. The slides were incubated for 90 min with the primary antibodies. The reaction was visualized by the Novolink polymer detection system (Novocastra Laboratories). Appropriate positive (tonsil for both: CD4 and CD8, placenta for ICAM) and negative controls (the primary antibody was replaced with normal mouse IgG at an appropriate dilution) were included for each case. The results of immunohistochemistry were evaluated by two independent pathologists who were blinded to the clinical data.

### 2.4. IHC Evaluation of ICAM-1

ICAM-1 expression in cancer nests was evaluated with the high-Score method. The score was calculated according to the commonly used formula (the percentage of cells with strong/moderate/weak expression multiplied by 3/2/1, respectively) [21]. Endothelial ICAM-1 expression was scored in each tumor as the proportion of ICAM-1-positive vessels and categorized into four subgroups [18].

### 2.5. Evaluation and Classification of CD4+ and CD8+ Cells

Five areas with the most abundant lymphocyte distribution (hot spots) were selected and micro-photographs were taken. The quantitative analysis was performed with Multiscan 14.2 software. The number of CD4+ and CD8+ cells was counted exclusively within cancer nests. For each case, the mean index of CD4+ and CD8+ cells per single high-power field was counted and then statistically analyzed.

### 2.6. qPCR in Samples Obtained with Microdissection

Separate populations of cancer and endothelial cells were harvested using LCM performed as follows. The selected, representative paraffin blocks were cut into 4 µm slices and mounted on slides with PEN-membrane (25 × 76 mm) (No. Cat. 11505158, Zeiss). Next, a brief staining protocol was applied, composed of deparaffinization in fresh xylene for 1 min twice followed by 100% ethanol for 1 min, 95% for ethanol 1 min, and 70% ethanol for 1 min. The slides then were transferred into distilled water for 2 min before staining with Hematoxylin for 2 min. Subsequently, slides were rinsed in distilled water until clear before undergoing dehydration in 70% ethanol for 1 min, 95% ethanol for 1 min, 100% ethanol for 1 min, and xylene for 1 min. Areas for microdissection were marked on the original hematoxylin-eosin–stained slide to include two targets, cancer nest and vessels in the surrounding stromal connective tissue. Microdissection was performed within the next 2 h with the PALM MicroBeam ZEISS Microscopy system. Tissue material was separately collected into AdhesiveCap (Item Number: 415190-9211-000, Zeiss).

Total RNA was extracted from the microdissected FFPE tissues (*n* = 118, two samples for each patient) using RNeasy FFPE Mini Kit (Qiagen) according to the manufacturer’s protocol. The quantity and quality of the isolated RNA were determined with NanoDrop 1000 UV Spectrophotometer (Thermo Scientific). Reverse transcription for the 118 microdissected samples was performed with iScript Advanced cDNA Synthesis Kit for RT-qPCR protocol (Bio-Rad).

qPCR to evaluate the expression of *EGF7* was performed using TaqMan Gene Expression Assay (Applied Biosystems) following the manufacturer’s protocol using the LightCycler 480 II system (Roche). The samples were analyzed in duplicates.

### 2.7. Statistical Analysis

The Shapiro–Wilk test and Leven’s tests were used to evaluate normality and equality of variances. Data for continuous variables are expressed as median with maximum and minimum values. As the assumptions of the tests were not met, statistical analyses were carried out using the Mann–Whitney test for quantitative variables. Furthermore, the Chi-square test and Fisher’s exact probability tests for comparison of qualitative variables were used. Overall survival curves were estimated by the Kaplan–Meier method and compared with the use of the two-sided log-rank test. *p*-values of <0.05 were regarded as significant in each of the analyses. All analyses were performed with the statistical software Statistica 13 (Stat Soft Inc.)

## 3. Results

### 3.1. Patient Population

The median age of the patients was 59 years (range 39–86), and the median duration of follow-up was 23.83 months (range 1.07–78.07). The 5-year disease free survival (DFS) rate was 20.35 %. The clinicopathological HGOSC data are presented in Table 2.

### 3.2. EGFL7 Expression at mRNA Level

Expression of *EGFL7* within the tumor microenvironment (epithelium and/or adjacent endothelium) was detected in 13 of 59 (22.03%) HGOSCs. 

We noted the increase in the PCR product only in a low proportion of samples; therefore, the initially planned quantitative analysis was not possible and the qualitative scoring (positive/negative) had to be applied. In detail, activation of *EGFL7* was detected in cancer epithelium (9/59, 15.25%), endothelium (8/59, 13.56%), or in both: cancer epithelium and adjacent endothelium (4/59, 6.78%). The presence of expression of *EGFL7* had no impact on progression free survival (PFS) and overall survival (OS) of the patients (*p* = 0.175 and *p* = 0.912 respectively). 

Association of clinicopathological HGOSC population data and *EGFL7* expression of cancer cells and endothelium are presented in Table 3 and Table 4, respectively.

No matter *EGFL7* was expressed in cancer epithelium, endothelium or in both, it had no impact on OS (*p* = 0.761, *p* = 0.849, *p* = 0.995, respectively).

### 3.3. ICAM-1 Expression

**Figure 1:**
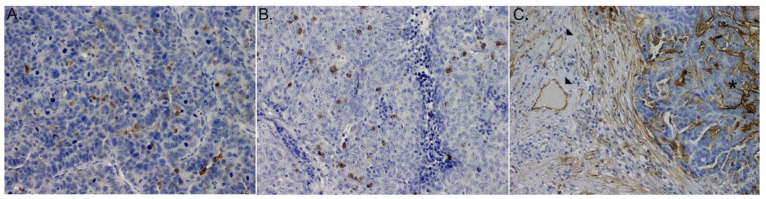
Microphotograph of immunohistochemical staining for: (**A**) (IE)CD4+ lymphocytes, (**B**) (IE)CD8+ lymphocytes, (**C**) ICAM-1 within primary HGSOCs: (stars) expression on cancer cells, (arrows) expression on immune cells.

We observed that ICAM-1 was expressed by both cancer and endothelial cells (Figure 1C). 

ICAM-1 expression in cancer cells was noted in 47 cases (79.66%) and in endothelial cells in 46 cases (77.97%); in 40 cases (67.8%) the expression was observed in both compartments. ICAM-1 expression in cancer cells was the score with the high-score method with a median expression level of 20 (range 0 to 200); 43 cases (91.48%) were scored as 1+ (median expression level of 20 range 5–90) and four (8.52%%) as 2+ (median expression level of 80 range 60–100).

Endothelial ICAM-1 expression was calculated as the proportion of positive vessels (median 18.75%, range 0%–94.12%) and then scored semi-quantitatively with a 4-tier system using the thresholds of 1%, 30% and 60%. Thus, 13 cases (22.03%) were scored as 0, 25 (42.37%) as 1+, 12 (20.34%) as 2+ and nine (15.25%) as 3+. 

There is no difference in the expression of ICAM-1 in the different clinicopathological subgroups (shown in Table 3). Moreover, no significant correlation was found between ICAM-1 expression and clinicopathological features furthermore Spearman rho was <0.3).

Based on the median ICAM-1 score in cancer cells (20), we dichotomized the group into a high expressing subgroup (22 cases, 37.29%) and a low expressing subgroup (37 cases, 62.71%). Similarly, the median proportion of ICAM-1-positive vessels (18.75%) was used to divide the high expressing subgroup (30 cases, 50.85%) and low expressing subgroup (29 cases, 49.15%). The clinicopathological characteristics related to ICAM-1 status of cancer and endothelial cells are depicted in Table 2 and Table 3, respectively. We observed no differences in OS or PFS between high and low ICAM-1 expression in either compartment (*p* > 0.05).

**Figure 2:**
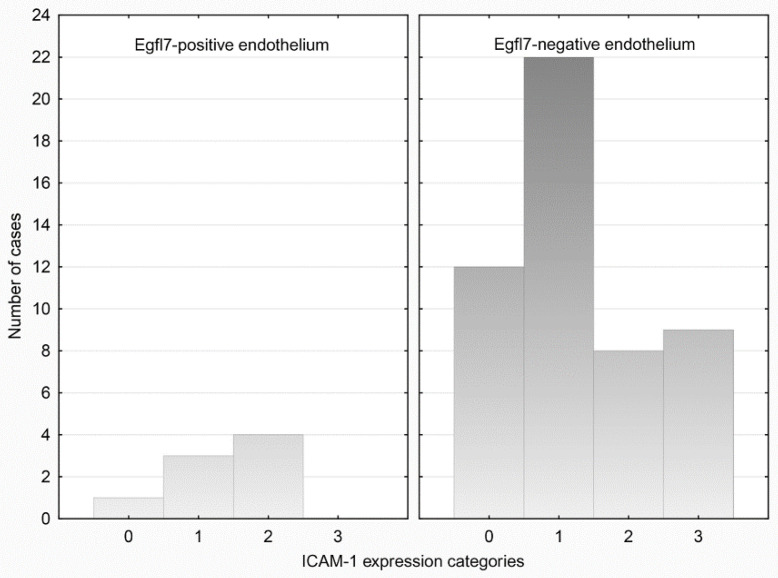
Categories of endothelial-ICAM-1 expression in relation to Egfl7 status of endothelial cells.

We observed no significant association between *EGFL7* and ICAM-1 expression in either compartment (*p* > 0.05). *EGFL7* expression in cancer cells was observed in 6/47 (12.77%) ICAM-1-positive and in 3/12 (25.00%) ICAM-1-negative tumors; ICAM-1 scores were also similar whether *EGFL7* was expressed or not. Analogously, *EGFL7* expression in endothelia was observed in 7/46 (15.21%) ICAM-1-positive and in 1/13 (7.69%) ICAM-1-negative cases; the proportions of ICAM-1 expressing vessels were also similar in both groups (Figure 2).

### 3.4. Immunohistochemistry for CD4+ and CD8+Cells

Both, CD4+ and CD8+ T cells were detected within cancer cell nests and sporadically in the mesenchymal stroma (Figure 1A,B). For purpose of this study intraepithelial (IE) CD4+ and CD8+ infiltrates were further evaluated. The median number of (IE)CD4+ and (IE)CD8+cells was 5 (range 0.33–26.33) and 12.33 (range 1.00–53) per single (HPF), respectively. 

### 3.5. Association of mRNA Egfl7 Status with (IE)CD4+ and (IE)CD8+ Cells

Further, we compared the number of (IE)CD4+ and (IE)CD8+ infiltrates between tumors with different *EGFL7*-status of cancer epithelium and endothelium. 

Tumors with *EGFL7*-positive cancer cells (*n* = 9) were less infiltrated with (IE)CD4+ and (IE)CD8+ lymphocytes compared to negative ones (*n* = 50) (median 1.67, range 0.33–12.33 vs. median 6.00, range 0.67–26.33, *p* = 0.022 and median 5.00 range 1.0 to 12.67 vs. 13.17 range 1 to 53, *p* = 0.04, respectively).

Tumors with *EGFL7*-positive endothelial cells (*n* = 8) were less infiltrated with (IE)CD4+ and (IE)CD8+ lymphocytes compared to negative ones (*n* = 51) (median 2.0, range 0.33–10.00 vs. median 6.00, range 0.33–26.33, *p* = 0.029 and median 5.00 range 1.0 to18.0 vs. 12.67 range 1 to 53, *p* = 0.031, respectively).

Tumors having simultaneously *EGFL7*-positive cancer and endothelial cells (*n* = 4) were less infiltrated with (IE)CD4+ and (IE)CD8+ lymphocytes compared to the negative ones (*n* = 55) (median 1.00 range 0.33–3.00 vs. median 5.67, range 0.33–26.33, *p* = 0.007 and median 1.83 range 1.0 to12.67 vs. 12.67 range 1 to 53, *p* = 0.013, respectively).

## 4. Discussion

*EGFL7* expression has been previously analyzed in one study on human ovarian cancer tissue samples derived from 177 patients [22]. In that study immunohistochemical staining for *EGFL7* was performed using formalin fixed paraffin-embedded tissue microarrays; 72 of 177 analyzed cases (40.7%) had tumors with serous histology, but information about grading was not specified [22]. High levels of *EGFL7* expression were noted in 23 of 72 (31.9%) serous cancers. Survival analysis performed for the entire cohort showed that the epithelial ovarian cancer patients having tumors with high *EGFL7* expression had a poorer DFS but similar OS to those with low *EGFL7* expression [22]. 

Here, we analyzed *EGFL7* mRNA transcripts in HGOSC using a different methodology, thus, the results of both studies are difficult to compare. 

Interestingly, our study supports the suggestion that *EGFL7* expression is frequent in epithelial ovarian cancers as we detected *EGFL7* mRNA transcripts in 13 of 59 (22.03%) HGOSC cases. Unfortunately, we did not manage to perform quantitative analyses of mRNA in microdissected tissue samples, thus, the impact of intensity of expression of *EGFL7* mRNA on OS and PFS was not assessable. However, the *EGFL7*-status of the tumor microenvironment (cancer epithelium and adjacent endothelium) had no predictive and prognostic value in the analyzed cohort of HGOSC patients. 

*EGFL7*-expression levels were found to correlate with a higher tumor grade in gliomas [23] and colon cancer [24], not influencing the prognosis. However, it conferred a poorer prognosis and higher metastatic score in hepatocellular carcinoma [25], but a better prognosis and absence of lymph node invasion in human breast cancer [26]. These ambiguous results indicate that *EGFL7* expression in human cancer needs to be carefully analyzed as *EGFL7* may play a complex role in cancer biology depending on cancer origin [23,24,25,26] and the source of *EGFL7* secretion: cancer cells, endothelium or both. 

Since it had been proven that *EGFL7* is plasma membrane-associated sialidase, which could accumulate in distant tissues from the producing cells [15], it is impossible to answer if the expression of *EGFL7* observed in OvCas [22] comes from activated cancer epithelium or up-regulated endothelial cells in the surrounding stroma. Therefore, we decided to analyze the impact of *EGFL7* on diapedesis using LCM technology, which enables harvesting the epithelial and endothelial cells directly to give histologically enriched cell populations for separate qPCR analyses [19].

We expected to detect *EGFL7* mRNA at least in endothelial cells as these reflect upregulation of *EGFL7* gene in activated cancer vessels due to neoangiogenesis and/or inflammatory process. The presence of *EGFL7* mRNA transcripts found in microdissected cancer cells indicates the endogenic activation of the *EGFL7* gene in human HGOSCs. To the best of our knowledge, this is the first study documenting the activation of *EGFL7* in human HGOSC. Interestingly the frequency of epithelial and endothelial activation of *EGFL7* was comparable. Further, ICAM-1 was also found to be expressed both on cancer epithelium and endothelium of adjacent stromal vessels, thus we decided to assess the correlation between *EGFL7* and ICAM-1 matched according to the source of origin.

Although it was suggested that *EGFL7* negatively regulates the expression of ICAM-1 in endothelial cells [17] we did not find a difference in the intensity of ICAM-1 expression between endothelium with different *EGFL7*-status. Similarly, a lack of difference in ICAM-1 expression was observed for cancer ICAM-1 expressing cells with different Egfl7-statuses.

Our understanding of the role of ICAM-1 in ovarian cancer development remains limited. The elevated expression of ICAM-1 in freshly isolated ovarian cancer cells [27] suggests that ICAM-1 expression may promote the malignant progression of ovarian cancer. Findings of studies on other malignant tumors suggest that ICAM-1 contributes to carcinogenesis by at least two mechanisms. It mediates the accumulation of inflammatory cells, which facilitates the instability of the tumor environment, triggers tumorigenesis, and maintains the release of trophic factors to enhance cancer cell survival [28,29]. Second, ICAM-1 partially mediates the invasive and metastatic potential of cancer cells [28,29,30,31,32]. These observations were not confirmed for our cohort of HGOSC patients as we found that cancer-ICAM-1 expression was not correlated with the FIGO stage and lacked prognostic significance.

To assess the potential impact of *EGFL7* on diapedesis we decided to compare intraepithelial immune infiltrates between tumors having *EGFL7* activated in cancer nests and/or adjacent vessels and those without *EGFL7* activation.

The presence of *EGFL7* mRNA transcripts in the epithelium and/or endothelium of tumor microenvironment was associated with a lower influx of adaptive immune effectors (CD4+ and CD8+ lymphocytes) into cancer nests. This provides direct information that in some parts, immune escape is achieved by limiting the influx of immune effectors into cancer tissue by activation of *EGFL7*. The role of epithelial and endothelial *EGFL7* should be further evaluated in HGOSCs. 

The weakness of the current study is the lack of quantitative assessment of *EGFL7*, the retrospective design and the small size of the cohort involved. Its strength lies in separate analyses of *EGFL7* transcripts in epithelium and adjacent endothelium of histologically homogenous group HGOSC tissue samples and consistency of the treatment of patients under uniform standards enabling the assessment of the prognostic significance of all the analyzed biomarkers.

## 5. Conclusions

Endogenic *EGFL7* activation in cancer cells and its upregulation in the endothelium are frequent in HGOSCs and could negatively impact diapedesis. *EGFL7* associated down-regulation of ICAM-1 was not observed. The function and predictive value of *EGFL7* mRNA transcripts should be evaluated in larger cohorts.

## Figures and Tables

**Table 1:** Antibody characteristics.

Antibody		Nr. Cat	Dilution	Expression
EGFL7	Santa Cruz	Santa Cruz sc-101349	1:200	Nuclear-cytoplasmic
ICAM	Abcam	[MEM-111] (ab2213)	1:400	Membranous
CD4	Millipore	IHCR2110-6	RTU	cytoplasmic
CD8	Millipore	IHCR2114-6	RTU	cytoplasmic

**Table 2:** The clinicopathological characteristics of HGOSC patients (*n* = 59).

Feature	Value
Age (years)	median 59 (range 39–86)
Preoperative CA125 (U/mL)	median 849 (range 6.8–25,000)
Ascites Yes/No (number)	38/21
Preoperative peripheral blood neutrocytes (G/L)	median 6.15 (range 3.27–10.99)
Preoperative peripheral blood lymphocytes (G/L)	median 1.5 (range 0.43–3.02)
FIGO I (number/percentage)	8/13.6%
FIGO II (number/percentage)	1/1.7%
FIGO III (number/percentage)	48/81.4%
FIGO IV (number/percentage)	2/3.3%
Residual disease: 0 cm (number/percentage)	8/13.6%
Residual disease: <1 cm (number/percentage)	22/37.3%
Residual disease: >1 cm (number/percentage)	29/49.1%
OS (months).	median 26.99 (range 1.07–78.07)

**Table 3:** The clinicopathological characteristics of HGOSCs population related to Egfl7 and ICAM-1 status of cancer cells.

Feature	Cancer Egfl7(+)	Cancer Egfl7(−)	*p*	Cancer ICAM-1(+)	Cancer ICAM-1(−)	*p*
Age (years) median (range)	60 (40–84)	58.5 (39–86)	0.626	58 (39–84)	62 (43–86)	0.309
Preoperative CA125 (U/mL) median (range)	660 (6.8–3000)	1000.8 (19.56–25,000)	0.204	837 (6.8–25,000)	1304 (174–8910)	0.337
Ascites Y/N number	6/3	32/18	1	32/15	6/6	0.315
Preoperative neutrocytes (G/L) median (range)	7.17 (5.38–8.19)	6.02 (3.27–10.99)	0.446	6.15 (3.27–10.99)	5.64 (4.99–8.55)	0.929
Preoperative lymphocytes (G/L) median (range)	1.465 (1.28–2.99)	1.5 (0.43–3.02)	0.732	1.49 (0.43–3.02)	1.8 (1.12–2.55)	0.244
FIGO I	1	7	0.882	6	2	0.830
FIGO II	0	1		1	0	
FIGO III	8	40		38	10	
FIGO IV	0	2		2	0	
Residual disease: 0 cm	1	7	0.962	7	1	0.311
Residual disease: <1 cm	4	18		19	3	
Residual disease: >1 cm	4	25		21	8	
Platin sensitive	7	27	0.081	16	2	0.462
Platin refractor/resistance	0	18		26	8	

**Table 4:** The clinicopathological characteristics HGOSCs population related to Egfl7 and ICAM-1 status of endothelial cells.

Feature	Endothelium Egfl7 (+)	Endothelium Egfl7 (−)	*p*	Endothelium ICAM-1 (+)	Endothelium ICAM-1 (−)	*p*
Age (years) median (range)	59 (40–80)	59 (39–68)	0.625	59.5 (39–86)	57 (43–80)	0.752
Preoperative CA125 (U/mL) median (range)	1026 (19.56–3012)	837 (6.8–25,000)	0.795	900 (6.8–25,000)	570 (88–9935)	0.605
Ascites Y/N number	3/5	36/16	0.119	29/17	9/4	0.754
Preoperative neutrocytes (G/l) median (range)	7.665 (3.69–8.51)	6.02 (3.27–10.99)	0.502	5.925 (3.27–10.99)	6.15 (5.3–8.89)	0.221
Preoperative lymphocytes (G/l) median (range)	1.63 (1.18–2.11)	1.43 (0.43–3.02)	0.394	1.5 (0.43–3.02)	1.4 (0.64–2.27)	0.991
FIGO I	1	7	0.914	5	3	0.456
FIGO II	0	1		1	0	
FIGO III	7	41		39	9	
FIGO IV	0	2		1	1	
Residual disease: 0 cm	1	2	0.705	6	2	0.659
Residual disease: <1 cm	2	20		19	3	
Residual disease: >1 cm	5	24		21	8	
Platin sensitive	6	28	0.081	15	3	1.000
Platin refractor/resistance	0	18		27	7	

## Data Availability

The datasets used and/or analyzed during the current study are available from the corresponding author on reasonable request.

## Abbreviations

*Egfl7*	Epidermal Growth Factor like domain 7
OvCa	Ovarian cancer
ICAM-1	Intercellular adhesion molecule-1
VCAM-1	Vascular cell adhesion molecule-1
LCM	Laser Capture Microdissection
HGSOC	High Grade Ovarian Serous Carcinoma
PDS	Primary debulking surgery
FIGO	The International Federation of Gynecology and Obstetrics
FFPE	Formalin-fixed paraffin-embedded
IHC	Immunohistochemical
DFS	Disease free survival
PFS	Progression free survival
OS	Overall survival
HPF	High-power field
IE	Intraepithelial
